# Introducing the Cardiovascular, metabolic and lipoprotein translation section of journal of translational medicine

**DOI:** 10.1186/1479-5876-10-203

**Published:** 2012-09-26

**Authors:** Nehal N Mehta

**Affiliations:** 1 Lab of Inflammation and Cardiometabolic Diseases, National Heart Lung and Blood Institute, Bethesda, MD, USA

## Abstract

Introducing the Cardiovascular, metabolic and lipoprotein translation section of journal of translational medicine.

## 

The *Journal of Translational Medicine* has focused on all areas of translational medicine derived from human experimentation to facilitate the communication between basic and clinical science. As the obesity, diabetes and cardiovascular disease epidemic continues to rise, especially with industrialization of developing countries [1], there is great need to focus on disseminating information between scientists and clinicians within this ‘cardiometabolic arena’. Despite aggressive control of cardiovascular disease risk factors, residual risk [2] related to untreated or undiscovered pathways demonstrate the need for intensive research and communication in this field.

Indeed the thrust to discover novel pathways for therapeutics within the cardiometabolic arena is accelerating rapidly with the continued advent of technologies in genomics [3], biological assays [4] and collaborative science [5]. T1 translation [6] strives to connect basic science research to clinical research and has a strong foundation with a long history of wet lab experimentation. Basic scientists have a rich array of forums and journals in which they can disseminate their findings. Additionally, T2 translation revolves around the goal of bridging research into everyday clinical practice and health decision making, and includes guideline development, meta-analyses, and systematic reviews. A wide number of journals which are geared toward practitioners exist to facilitate communication within these fields. Finally, T3 includes dissemination and implementation research which includes policy-making and population-focused efforts, and many governmental agencies and publication forums are available for these efforts as well.

However, groups performing cardiometabolic and lipoprotein-based multi-disciplinary, translational research between the T1 and T2 phases often struggle to find an appropriate target journal to publish their findings because of the mixed content of basic and clinical research. This mismatch often delays publication of important findings due to the combination of multiple submissions to basic science and clinical journals. In order to increase the speed at which discoveries move from the lab into the clinic, ultimately improving the health of populations, fast and efficient communication is essential to keep up with these moving targets. Therefore, we here introduce the section of Cardiovascular, metabolic and lipoprotein translation within the *Journal of Translational Medicine,* which is devoted to the rapid publication of research papers within these fields.

This section aims to speed mechanistic understanding, development of novel diagnostics and potential therapeutic targets within cardiovascular, metabolic diseases and lipoprotein diseases. We are open to experiments involving cellular models, animal models, observational studies and clinical trials that are distinguished for their novelty in approach, design or indication, timeliness, and unique ability to translate laboratory concepts from the bench to the bedside. Highest priority will be given to those manuscripts which are specifically focused on cardiovascular, metabolic and lipid disorders which enrich understanding of disease mechanism, novel therapeutics and state-of-the-art scientific technique.

We invite your group to consider publishing your findings within this exciting new section which will be overseen by a talented group of Editorial Board members within cardiovascular medicine, lipoprotein biology, endocrinology, metabolism, animal biology, basic science and human genomics.
